# Partial purification and characterization of 2, 4-diacetylphloroglucinol producing *Pseudomonas fluorescens* VSMKU3054 against bacterial wilt disease of tomato

**DOI:** 10.1016/j.sjbs.2021.02.073

**Published:** 2021-03-04

**Authors:** Perumal Suresh, Govintharaj Varathraju, Vellasamy Shanmugaiah, Khalid S. Almaary, Yahya B. Elbadawi, Ayman Mubarak

**Affiliations:** a Department of Microbial Technology, School of Biological Sciences, Madurai Kamaraj University, Madurai 625 021, Tamil Nadu, India; b Department of Botany and Microbiology, College of Science, King Saud University, P.O. 2455, Riyadh 11451, Saudi Arabia

**Keywords:** *Pseudomonas fluorescens*, DAPG, *Ralstonia solanacearum*, High content screening and detached leaf assay

## Abstract

We find out the antimicrobial potential of partially purified 2,4-diacetylphloroglucinol (DAPG) against *Ralstonia solanacearum* and fungal plant pathogens isolated from tomato rhizobacterium *Pseudomonas fluorescens* VSMKU3054. The present study is mainly focused on the control of wilt disease of tomato by our isolate VSMKU3054 and DAPG. The cell free culture filtrate of *P. fluorescens* VSMKU3054 was significantly arrested the growth of *R. solanacearum* and fungal pathogens such as *Rhizoctonia solani*, *Sclerotium rolfsii*, *Macrophomina phaseolina* and *Fusarium oxysporum* compared to control. The existence of DAPG from the crude metabolites of *P. fluorescens* VSMKU3054 was confirmed on TLC with Rf value 0.34, which is coincide with that of authentic phloroglucinol. The partially purified DAPG exhibited much higher activity against *R. solanacearum* at 30 µg/ml than the fungal plant pathogens compared to control. The antimicrobial partially purified compound was identified as DAPG by UV, FT-IR and GC–MS analysis. The percentage of live cells of *R. solanacearum* when supplemented with DAPG at 30 µg/ml, significantly controlled the living nature of *R. solanacearum* up to 68% compared to tetracycline and universal control observed under high content screening analysis. The selected isolate *P. fluorescens* VSMKU3054 and DAPG significantly controlled wilt disease of tomato up to 59.5% and 42.12% on 3rd and 7th days compared to positive and negative control by detached leaf assay. Further, in silico analysis revealed that high interaction of DAPG encoding protease with lectin which is associated with *R. solanacearum*. Based on our findings, we confirmed that *P. fluorescens* VSMKU3054 and DAPG could be used a potential bio inoculants for the management of bacterial wilt disease of tomato.

## 1 Introduction

Bacterial wilt disease of tomato (*Lycopersicon esculentum* L.,) caused by *Ralstonia solanacearum*, is one of the most economically important bacterial disease among other bacterial pathogens. *R. solanacearum* is an endemic pathogen in most of the warm environmental conditions (Hayward, 1991, Elsayed et al., 2020). The yield loss of tomato by the action of *R. solanacearum* is up to 60 to 100% (Popoola et al., 2015). *R. solanacearum* can infect more than 200 plant species, especially Solanaceae, Musaceae families and other crops (Li et al., 2014, Mohammed et al., 2020). Bacterial wilt pathogen *R. solanacearum* enter into the root system and xylem vessels through cuticle, stomata and other natural openings (Hayward, 1991, Denny, 2006, Lowe-Power et al., 2018) This pathogen initially infect young leaves of tomato and after some duration, the leaves of tomato will become flaccid, wilting and hole plant will become yellow (Vanitha et al., 2009: Chaudhry and Rashid, 2011). During the development of disease, *R. solanacearum* secretes various virulent genes such as extracellular polysaccharide, cell wall degrading enzymes and type III secreted effectors molecules. (Kao et al., 1992, Zhou et al., 2012, Meng, 2013, Coll and Valls, 2013). In addition to that *R. solanacearum* highly persistent and vigorously colonizing inside the root system by up regulation of genes involved in response to root exudates and low oxygen condition in agriculture field, degradation pathways against plant defense molecules, the adaptation to the nutrient underprivileged xylem environment (Brown and Allen, 2004, Colburn-Clifford and Allen, 2010, Lowe et al., 2015, Lowe-Power et al., 2018).

Hence, in this context, there is an urgent need for the control of bacterial wilt disease of tomato without causing any environmental pollution and health hazards. In recent scenario, various strategies were developed to control wilt disease of tomato from microbial origin instead of using chemical pesticide, fungicide and fertilizers. Moreover, there is no successful chemical to control bacterial wilt disease of tomato (Zhou et al., 2012). In recent days, fluorescent pseudomonads (FPs) have drawn much attention worldwide for the control of fungal and bacterial pathogens (Haas and Défago, 2005, Couillerot et al., 2009, Mohammed et al., 2020). The current interest has been increased using beneficial microorganisms such as *Pseudomonas* spp (Sakthivel and Gnanamanickam, 1987, Shanmugaiah et al., 2010, Nithya et al., 2020, Basu et al., 2021), *Bacillus* spp (Shanmugaiah et al., 2008, Arasu et al., 2016, Rais et al., 2017, Khan et al., 2020, Al-Dhabi et al., 2020), *Streptomyces* spp (Prabavathy et al., 2006, Harikrishnan et al., 2014) and *Trichoderma* spp (Mathivanan et al., 2005., Shanmugaiah et al., 2009, Sood et al., 2020). Among different, beneficial microorganisms, FPs is a predominant rhizospheric microorganism and easily adapts various environmental conditions. Because, FPs have the ability to control plant disease by two different mechanisms such as direct and indirect (Compant et al., 2005, Nithya et al., 2020). FPs are produced wide range antimicrobial metabolites such as Phenazine (Shanmugaiah et al., 2010, Nithya et al., 2020), 2,4-diacetylphloroglucinol (DAPG) (Velusamy et al., 2006, Zhou et al., 2012), pyrrolnitrin (De Souza and Raaijmakers, 2003), pyoluteorin (Hu et al., 2005), Hydrogen cyanide (Defago and Haas, 1990) and lytic enzymes (Zhou et al., 2012, Mohammed et al., 2020).

DAPG is one of the most important polyketide antimicrobial compounds produced by different FPs (Tada et al., 1990, Velusamy et al., 2006, Gong et al., 2016). *Pseudomonas* sp. is used to control different soil-borne plant pathogens like bacteria, fungi, nematodes and other pathogens by the production DAPG (Sharifi-Tehrani et al., 1998, De Souza et al., 2003, Zhou et al., 2012). Naturally, the DAPG have a key role in biological control of plant diseases such as wheat (*Gaeumannomyces graminis* var. *tritici*), black root rot of tobacco (*Thielaviopsis basicola*), and damping-off of sugar beet (*Pythium ultimum*) by *P. fluorescens* (Keel, 1992, Harrison et al., 1993, Shanahan et al., 1992, Marchand et al., 2000). Velusamy et al., (2006) reported that bacterial blight pathogen was inhibited by DAPG in greenhouse conditions and the results were exhibited 59 to 64% efficiency. DAPG also involves herbicidal activity on sugar cane and cereals and other crops (Keswani et al., 2020). In this context, several studies demonstrated that DAPG have antifungal metabolites but only few reports are explored against *Ralstonia solanacearum*.

The *phlD* encoding gene is used for the detection of 2, 4-diacetyl phloroglucinol from rhizosphere obtaining *Pseudomonas* spp. *phlD* is responsible for the production of monoacetylphloroglucinol (MAPG). Similarly, *phlA*, *phlB*, and *phlC* are involved in the conversion of MAPG to 2,4-DAPG (Almario et al., 2017, Zhao et al., 2020). The economically important soil-borne fungal pathogens such as *Fusarium oxysporum, R. solani, M. phaseolina,* and *S. rolfsii* were successfully controlled by Phenazine derivatives (Nithya et al., 2020), 2, 4-DAPG (Velusamy et al., 2006, Zhou et al., 2012) producing *Pseudomonas* spp. Many potential antimicrobial compounds obtained from *Pseudomonas* spp and its mechanism in *in vitro* and *in vivo* conditions are not explored properly. Hence, molecular docking is an effective tool in drug designing used for structure prediction of specific ligand–protein and protein–protein interaction (Kataki and Saikia, 2015).

Alternative strategy is essential for the control of plant disease without creating any health hazards and environmental problems. Hence, biological control is a suitable method for suppression of bacterial wilt disease and increasing crop production for sustainable agricultural system by FPs from rhizosphere origin. The current study was focused on the following objectives i) To extraction of metabolite from *P. fluorescens* VSMKU3054 ii) characterization of partially purified DAPG by different spectral studies iii) To study, the antimicrobial potential of *R. solanacearum* and different fungal pathogens iv) Biocontrol potential of *P. fluorescens* VSMKU3054 and DAPG against bacterial wilt disease of tomato v) To study in silico analysis of DAPG encoding gene towards *R. solanacearum.*

## 2 Materials and methods

### 2.1 Bacterial strains and culture conditions

*Pseudomonas fluorescens* VSMKU3054 was isolated from tomato rhizosphere and the accession no. is MH443348. *P. fluorescens* was grown in King’s B agar (KBA) medium at 37 °C for 24 h and fluorescent colonies were observed under UV illuminator at 360 nm. The *Ralstonia solanacearum* was grown in Kelmen TZC medium at 28 °C for 48 h. The cultures were maintained in 30% glycerol stock at −20 °C for further experiments.

### 2.2 Effect of cell-free culture filtrate against *R. Solanacearum* and fungal pathogens

The isolate VSMKU3054 was grown (100 ml) on King’s B broth in 250 ml conical flask at 37 °C on a rotatory shaker at 120 rpm for 24 h. At 10,000 rpm at 4 °C for 20 min, the culture was centrifuged and sterilized by passing it through Millipore membrane filter paper. The cell-free culture filtrate was tested against *R. solanacearum* and fungal pathogens such as *R. solani, S. rolfsii, M. phaseolina,* and *F. oxysporum* by well diffusion method (Shanmugaiah et al., 2006). Nine mm of four wells were made on Nutrient Agar (NA) and PDA using sterile cork borer. The different volumes (25, 50, 75, and 100 μl) of cell-free filtrate were added to NA for *R. solanacearum* and PDA for fungal pathogens. The fresh *R. solanacearum* (10^7^ CFU/ml) was spread on the NA before making the well, similarly 9 mm of fresh fungal pathogen was placed on the PDA. The medium with comparable concentration was poured into both NA and PDA petri plates and fresh pathogens were inoculated as control. The Petri plates were incubated at 28 ± 2 °C for 4 days. The inhibitory effect of cell culture filtrate was carried out in randomized block design triplicates and replicated three times. The bacterial colony inhibition zone and the mycelial growth of the pathogens were measured in mm.

### 2.3 Extraction VSMKU3054 crude metabolites and TLC

The isolate VSMKU3054 was cultured in 2L of King’s B broth at 37 °C on shaker at 120 rpm for 24 h. The culture was centrifuged at 4 °C for 20 min at 10,000 rpm. The collected cell free filtrate was extracted four times with an equivalent volume of ethyl acetate. The extraction was air-dried two times by rotary evaporation at 50 °C. The crude metabolites were analyzed on thin layer chromatography (TLC) with solvent system chloroform: methanol (9:1). Five different spots were observed on TLC; among them one spot was scrapped with Rf value 0.34 which is coincide with that of authentic phloroglucinol (Sigma, Mumbai, India).

### 2.4 Antimicrobial activity of partially purified DAPG metabolites

The partially purified DAPG antimicrobial effect on *R. solanacearum* and fungal pathogens such as *R. solani, S. rolfsii, M. phaseolina*, and *F. oxysporum* was tested by well diffusion method (Shanmugaiah et al., 2010). Different concentration of partially purified DAPG was added to the agar well ranging from 5 to 160 µg/ml. As a positive control, the commercial antibiotic tetracycline and carbendazim were used. The varying inhibition zone levels were observed. Mycelial discs of *R. solani, S. rolfsii, M. phaseolina,* and *F. oxysporum* were cut from a 5-day-old culture and placed at the center of the petri plates. For each treatment triplicate plates were maintained. At room temperature (28 ± 2 °C) the plates were incubated for up to 5 days, and the mycelial radial growth of fungal pathogens was recorded.

Likewise, the one-day-old culture of *R. solanacearum* was swabbed on the nutrient agar surface using sterile cotton buds. Aqueous solutions of partially purified DAPG and tetracycline were prepared separately from 5 to 160 µg/ml concentrations and then filter sterilized. Four wells were made in each plate using sterile cork borers, and the above said concentration of aqueous solution of DAPG and Tetracycline was introduced to respective well. Sterile water was placed in the control wells. For each treatment, triplicate plates were maintained at room temperature and the zone of inhibition was assessed after 48 h.

### 2.5 Characterization of VSMKU3054 DAPG metabolites

The scrapped DAPG metabolites were characterized by UV–Vis spectrophotometer with an absorbance range between 200 and 400 NM. FT-IR spectrum was recorded in 400–4000 cm^−1^ and the DAPG were mixed with KBr (Shimadzu). The GC–MS analysis was performed for DAPG metabolites extracted from *P. fluorescens* VSMKU3054 with standard phloroglucinol. Conversion and selectivity were determined by Agilent 7820A GC using an HP-5 column of 30m length and highly pure nitrogen (99.999%) as carrier gas with an FID detector. The initial temperature of the oven was kept at 50 °C and the final temperature was 10 °C/min to 280 °C. The products detected in the final reaction mixture have been confirmed with an Agilent GC–MS instrument 5890A model using 30 m long HP-5 column and high purity helium (99.99%) as carrier gas.

### 2.6 Live and dead assay

Live and the dead assay was performed to validate our partially purified metabolite DAPG against *R. solanacearum* under High Content Screening (HCS). Briefly, *R. solanacearum* (1x10^8^ CFU/ml) treated with partially purified DAPG and tetracycline at 30 µg/ml and incubated for 8 h at 28 ± 2 °C. Acridine orange and propidium iodide were stained on *R. solanacearum* cells (Paret et al., 2010). The cells were analyzed under a 40X magnification HCS imaging microscopy microscope (Perkin Elmer Operetta), and the image analysis was carried out using the program Harmony 3.0.

### 2.7 Detached leaf assay

Detached leaves assay was performed using five weeks old tomato leaves without any wounds and selected leaves were surface sterilized by soaking in a 1% sodium hypochlorite solution for 2 min. These leaves were then thoroughly rinsed with sterile distilled H_2_O, leaves were dried for 5 min. For treatments, the overnight grown *P. fluorescens* VSMKU3054 and *R. solanacearum* cell density was adjusted to 1x10^8^ CFU/ml. Each treatment consisted of one detached leaf. As soon as the surface of detached leaves dried, a 50 µl of both bacterial cell suspensions was dropped for the inoculation in detached tomato leaves. Similar volumes of 30 µg/ml of partially purified DAPG and commercial tetracycline was injected in detached leaves of tomato. Distilled H_2_O was used as negative control for the comparison of other treatments. A total of three replications were maintained for all the experiments. All the treated detached leaves of tomato were placed on the 0.5% agar surface in Petri dishes. All plates were incubated at 28 ± 2 °C for 1, 3, and 7 days in light/dark conditions (16/8 h) and humidity was maintained. After seven days of incubation, disease severity was assessed (Afroz et al., 2009). The disease scale was calculated and the relative AUDPC (Area under the disease progress curve) percentage was determined as described by Lee et al., (2012).

### 2.8 Molecular docking of DAPG gene encoding protein

DAPG encoding gene has been identified in *P. fluorescens* VSMKU3054. The sequenced and further analysis of the docking was carried out. To understand the activity of DAPG encoding protein, the molecular docking analysis was carry out using the Auto Dock 4.2 program with DAPG three-dimensional (3D) protein structure (Morris et al., 2009). The 3D structure was designed using Modeller 9v2 software. The ligand structure was selected as a target for molecular docking studies, based on the previous studies.

### 2.9 Preparation of receptor and ligand

The protein molecule was extracted from the database of protein databases (PDB) and properly tested using Protein Structure Analysis (PROSA) (Wiederstein and Sippl, 2007), 3D (Luthy et al., 1992) and Ramachandran plot verification. The SWISS PDB viewer allowed energy minimization, hydrogen atoms addition, water molecules removal, and eventually Kollman charges were allocated and converted to PDBQT. The arrangement of proteins *R. solanacearam* was recovered from PDB. The system had been well prepared for further study of the docking. The water molecules were eventually extracted and the gasteiger charges and non-polar hydrogens were added to the ligand.

### 2.10 Molecular docking

The blind docking method identified the interacting residues and the target protein binding site (Sali and Blundell, 1993). The grid map of the receptors was created based on the whole protein and the grid points were set at 40 × 40 × 40 Å with points separated by 0.597 Å. The Lamarckian Genetic Algorithm (LGA) was used with the parameters of 10 docking experiments, 150 population size, and 2,500,000 maximum number of energy evaluations, 27,000 maximum number of generations, 0.02 mutation rate, and 0.8 crossover rate. Additionally, the best complexes were selected based on the lowest free binding or docking energy and saved as PDB. Eventually, the conformations were analyzed and visualized through LigPlus and PyMOL, with the aid of two programs.

## 3 Results

### 3.1 Antimicrobial potential of cell-free filtrate of *P. Fluorescens* VSMKU3054 against *R. Solanacearum* and phytofungal pathogens

The inhibition potential of cell-free culture (CFC) filtrate of *P. fluorescens* VSMKU3054 has shown significant antagonistic activity against *R. solanacearum* with the zone of inhibition of 22.33 mm (25 µl). Similarly, CFC of *P. fluorescens* VSMKU3054 (25 µl) has shown effective antagonistic activity against *R. solani* and *F. oxysporum*. A remarkable antagonistic activity against *S. rolfsii* and *M. phaseolina* was showed with 75 µl of CFC. Besides the growth of fungal mycelium, it was also significantly inhibited by CFC of *P. fluorescens* VSMKU3054 as compared to control (Table. 1).

**Table 1:** Effect of cell free culture filtrate of *P. fluorescens* VSMKU3054 against plant pathogens (mm).

Phytopathogns	Cell free culture filtrate of VSMKU3054 (µl)			
	25	50	75	100
*R. solanacearum*	24.67 ± 1.25	25 ± 0.82	25.67 ± 0.47	28 ± 1.41
*R. solani*	20. 67 ± 0.94	23.33 ± 1.24	23. 67 ± 1.24	24 ± 1.63
*S. rolfsii*	12.33 ± 0.47	14 ± 0.81	15. 67 ± 0.47	16. 67 ± 0.94
*M. phaseolina*	18.33 ± 1.52	19. 33 ± 0.57	21. 00 ± 1	22.67 ± 1.52
*F. oxysporum*	21.33 ± 0.47	22. 67 ± 0.47	23. 67 ± 0.47	25 ± 1.41

Values are calculated and expressed in triplicates with ± SEM.

### 3.2 Extraction of crude metabolites and TLC

**Fig. 1:**
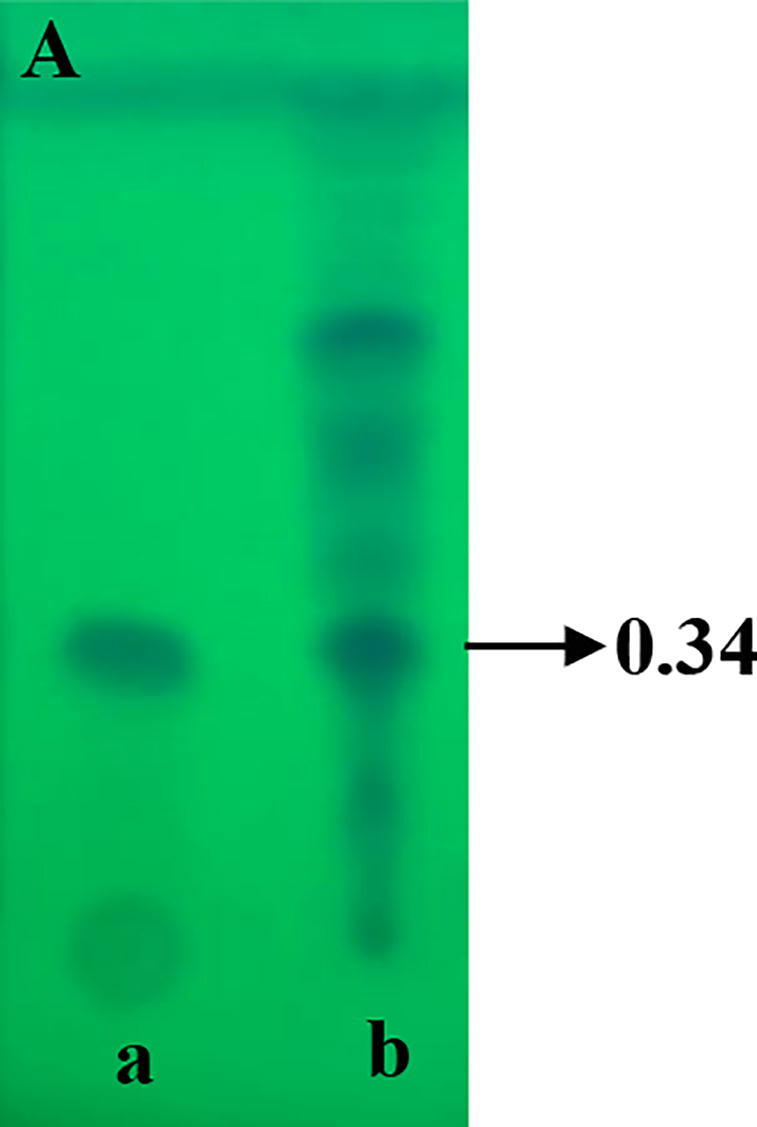

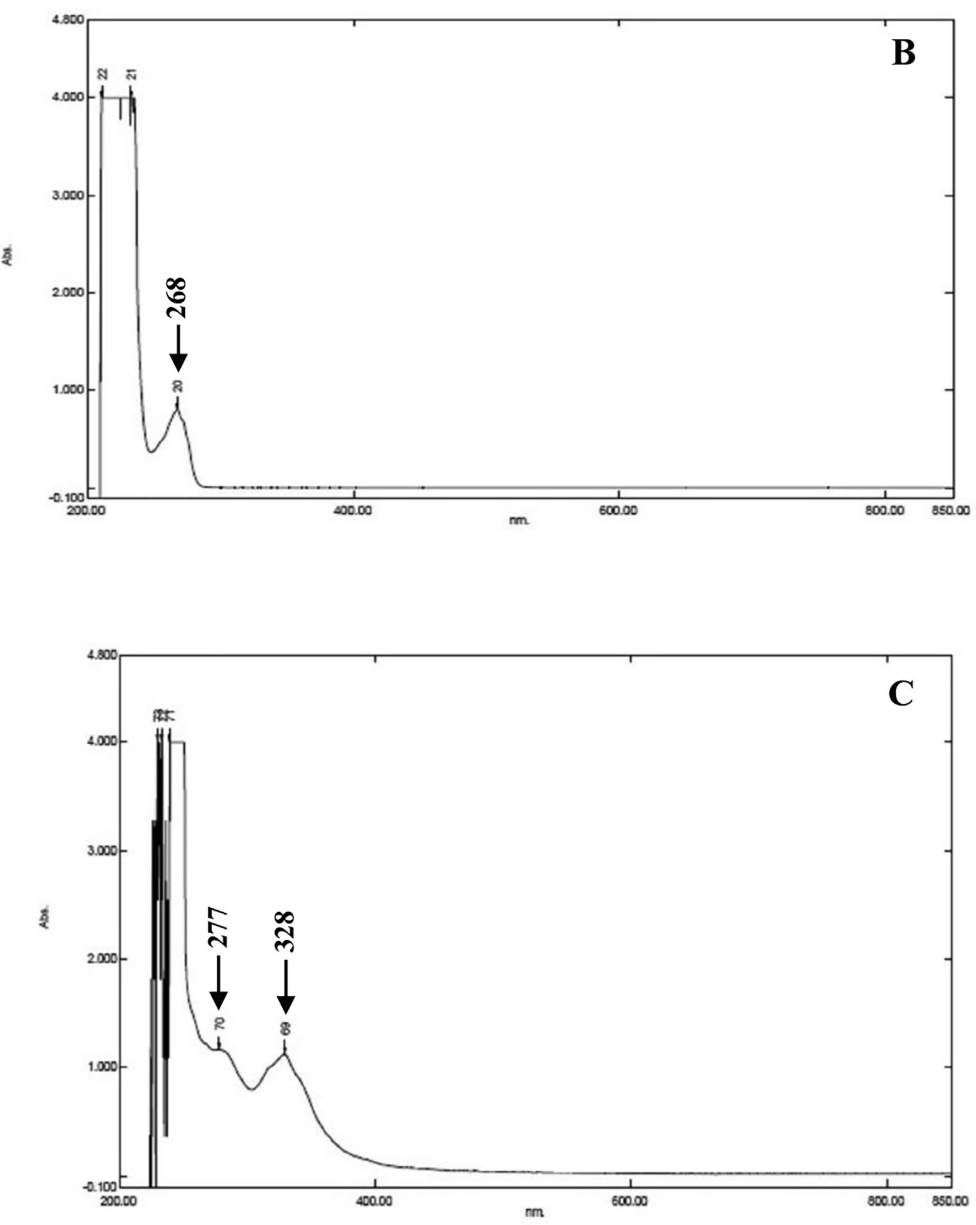

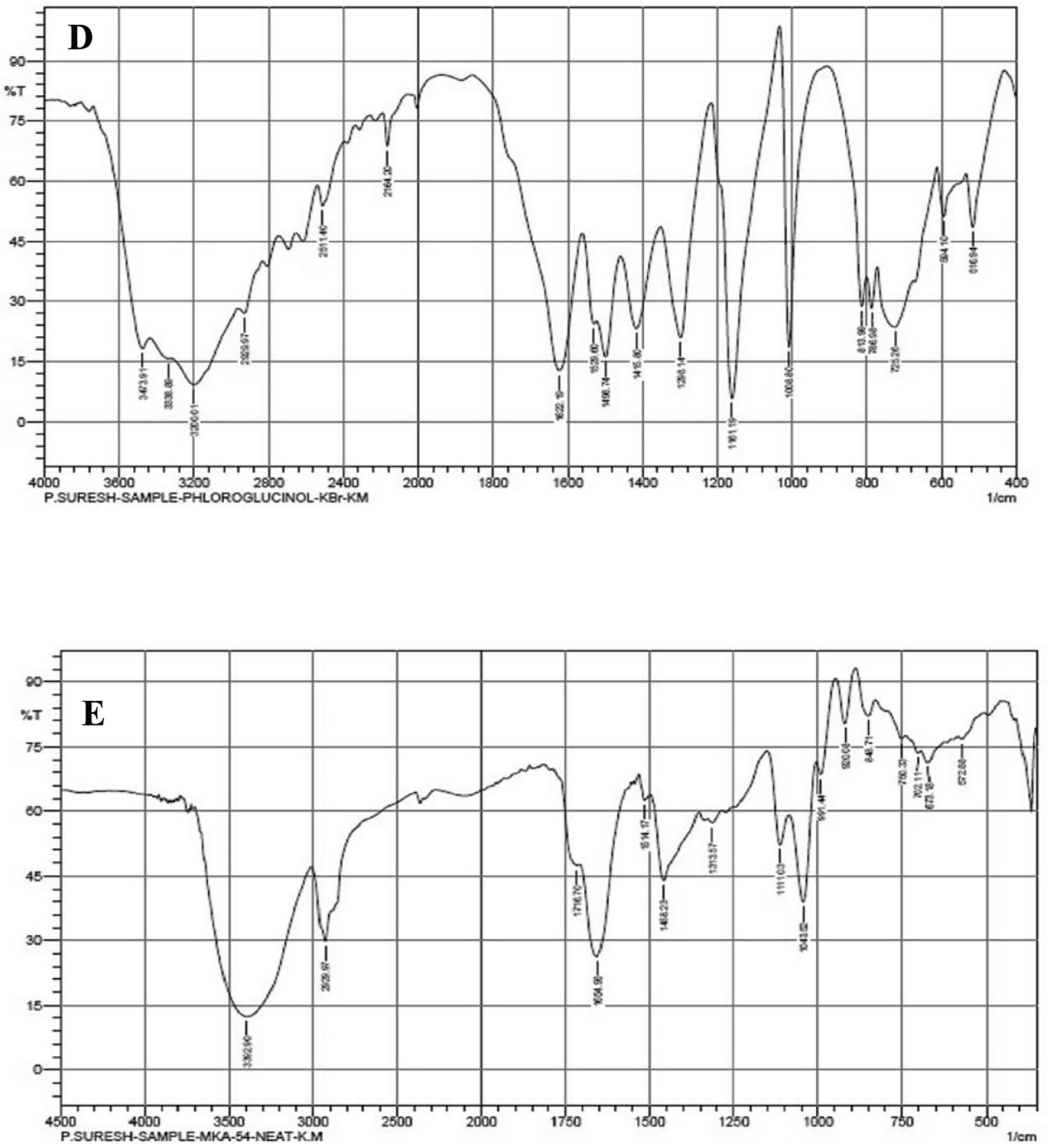

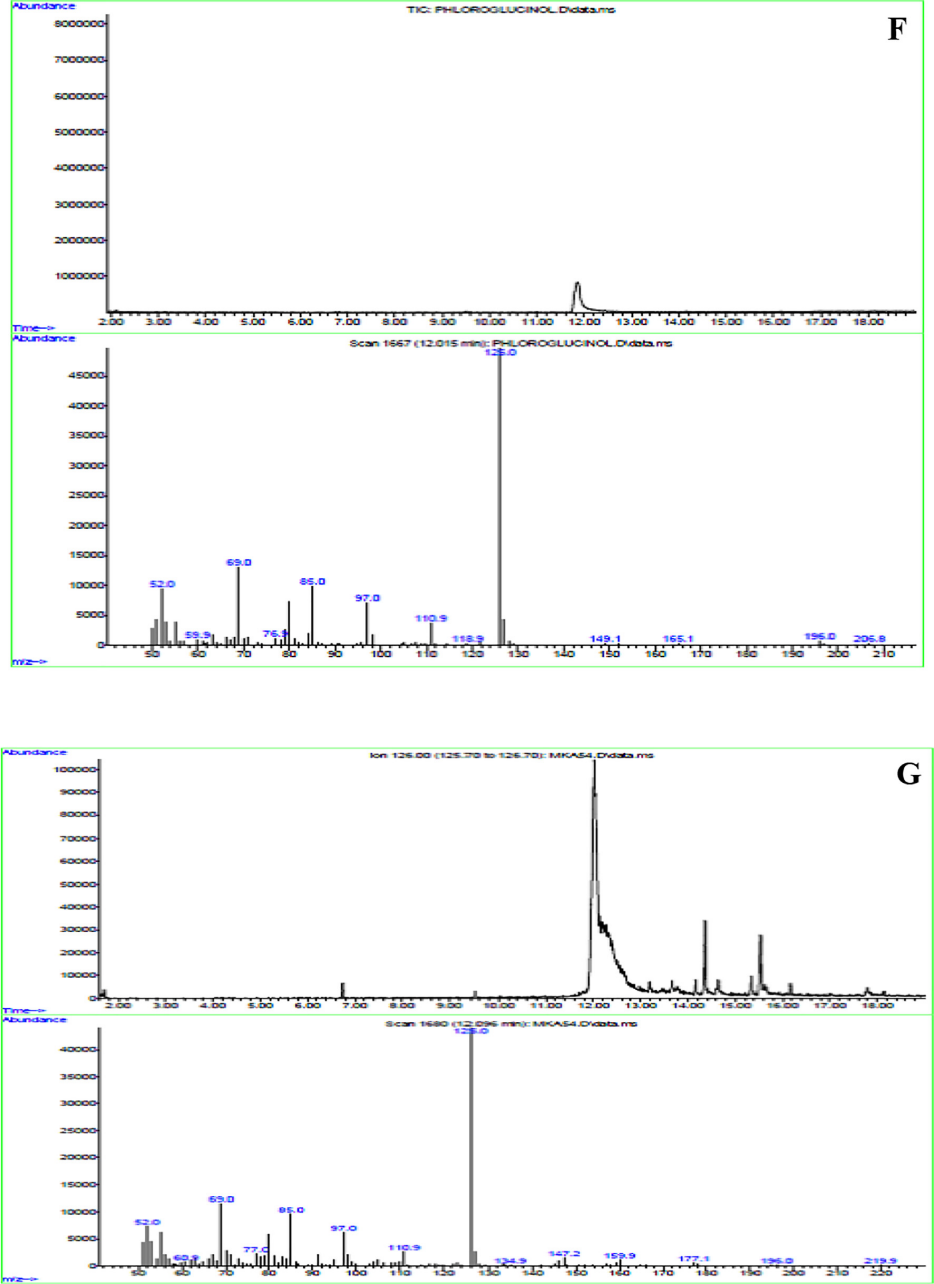
**Characterization of DAPG antimicrobial metabolites from *P. fluorescens* VSMKU3054:** A. TLC (a - Phloroglucinol & b - Crude), B. UV–Vis Spectrum of Phloroglucinol, C. UV – Vis Spectrum of *P. fluorescens* VSMKU3054, D. FTIR - Spectrum of Phloroglucinol, E. FTIR - Spectrum of *P. fluorescens* VSMKU3054, F. GC–MS – Phloroglucinol and G. GC–MS – *P. fluorescens* VSMKU3054.

The crude metabolites of *P. fluorescens* VSMKU3054 were extracted from the cell-free culture filtrate. The crude metabolites were analyzed by TLC, the R_f_ values obtained from TLC include 0.34, 0.43, 0.58, and 0.71, in which 0.34 corresponds to the authentic phloroglucinol (Fig. 1A).

### 3.3 Antimicrobial activity of partially purified DAPG against phytopathogens

The DAPG metabolites from *P. fluorescens* VSMKU3054 was showed remarkable antimicrobial activity against *R. solanacearum* at 30 µg/ml as compared to control (Table 2). Fungicidal activity of partially purified DAPG metabolites against fungal pathogens such as *R. solani, S. rolfsii,* and *M. pheaseolina* has shown significant growth inhibition at a concentration of 90. Whereas the DAPG metabolites at 150 µg/ml was showed remarkable activity against *F. oxysporum* as compared to control (Table 3).

**Table 2:** Effect of partially purified DAPG against *R. solanacearum* (mm).

S. No	Concentration(µg/ml)	*R. solanacearum*	Tetracycline
1.	**5**	12 ± 1	–
2.	**10**	17.33 ± 1.55	9.83 ± 1.04
3	**15**	19.67 ± 1.53	11.63 ± 0.35
4.	**20**	21.33 ± 0.58	13.37 ± 0.71
5.	**25**	22 ± 1	15.27 ± 0.7
6.	**30**	23 ± 1	17.13 ± 0.23

– indicates negative reaction; Values are calculated and expressed in triplicates with ± SEM.

**Table 3:** Effect of partially purified DAPG metabolites against fungal phytopathogens (mm).

Conc. of crude metabolites (µg/ml)	*R. solani*	*S. rolfsii*	*M. phaseolina*	*F. oxysporum*
**10**	11.5 ± 1.32	6.93 ± 0.45	8.3 ± 0.36	5.17 ± 0.72
**20**	12.23 ± 0.68	7.6 ± 0.4	8.83 ± 0.32	5.6 ± 0.35
**30**	12.77 ± 0.40	9.47 ± 0.61	9. 67 ± 0.31	6.13 ± 0.23
**40**	13.43 ± 0.51	9.53 ± 0.50	10.43 ± 0.55	8.17 ± 0.74
**50**	13.6 ± 0.36	9.97 ± 0.35	11.17 ± 0.76	9.33 ± 0.70
**60**	13.77 ± 0.21	10.63 ± 0.32	12 ± 0.87	9. 67 ± 0.35
**70**	13.97 ± 0.058	10.87 ± 0.23	13.7 ± 0.6	12.47 ± 0.32
**80**	14.63 ± 0.351	11.47 ± 0.61	14.57 ± 0.4	13.13 ± 0.25
**90**	23.6 ± 0.6	24.27 ± 0.64	22.77 ± 0.35	13. 67 ± 0.29
**100**	24.5 ± 0.56	26.13 ± 0.42	23 ± 0.36	14.3 ± 0.62
**110**	25.43 ± 0.49	26. 67 ± 0.25	23.93 ± 0.45	14.6 ± 0.4
**120**	26.17 ± 0.32	27.03 ± 0.15	24.37 ± 0.31	15.43 ± 0.4
**130**	26.93 ± 0.31	27.23 ± 0.32	25.17 ± 0.32	16.77 ± 0.32
**140**	27. 67 ± 0.75	27.7 ± 0.3	26 ± 0.62	17.83 ± 0.21
**150**	28.43 ± 0.51	28.5 ± 0.3	27.23 ± 0.4	18.87 ± 0.50
**160**	29.4 ± 0.35	29.53 ± 0.42	29.17 ± 0.49	20.1 ± 0.26

Values are calculated and expressed in triplicates with ± SEM.

### 3.4 Characterization of partially purified DAPG compound

UV spectrum of DAPG metabolites was showed peaks at ʎ_max_ 277.40 and 328.60 and the standard phloroglucinol showed a similar peak at ʎ_max_ 268 as shown in Fig. 1. FT-IR spectra analysis of DAPG has shown wavelength at 3392.9, 2929.9, 1716.7, 1654.9, 1514.1, 1458.2, 1313.5, 1111.0, 1043.5 and 991.4 cm^−1^ related peaks were observed against standard phloroglucinol. The DAPG compound shown the functional groups such as H-bonded OH, aromatic ring, C–H groups in C–CH3compound, April carbonyl compounds, C–OH in alcohols, ethers, acid esters (Fig. 1 B-E). The same retention time (12.096 and 12.015 min) was observed for both DAPG and standard phloroglucinol in GC–MS analysis with similar molecular mass (126 *m*/*z*) (Fig. 1 F, G).

### 3.5 Live and dead assay

**Fig. 2:**
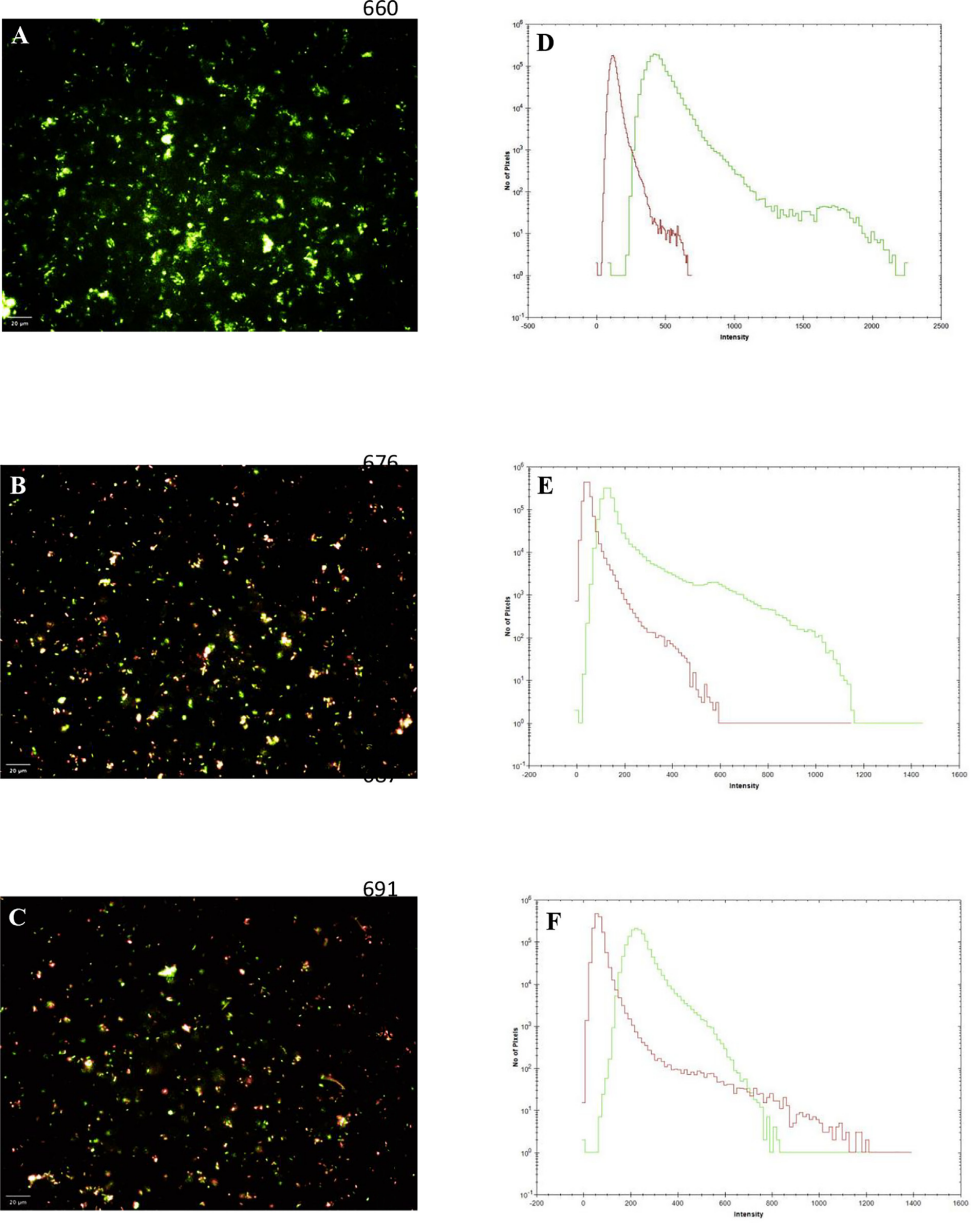
**Live/Dead assasy in high content screening analysis of DAPG extracted from *P. fluorescens* VSMKU3054 against *R. solanacearum***: Green colour considered as live cells and red colour as dead cells. A - *R. solanacearum*, B - *R. solanacearum +* tetracycline at 30 µl/ml and C - *R. solanacearum* + DAPG at 30 µl/ml, D, E and F - graph showed representative histogram based on the intensity of live and dead cells (A, B and C).

The cell viability assay was performed for examining the viability of *R. solanacearum* in response to *P. fluorescens* produced DAPG antimicrobial compounds. In control (*R. solanacearum*) 91% of the cells were stained green, which specified the highest proportion of live cells. *R. solanacearum* treated with tetracycline at 30 µg/ml has shown only 24% of live cells have appeared green in color. Whereas *R. solanacearum* when treated with DAPG at 30 µg/ml has shown significant inhibition of *R. solanacearum* with 32% of live cells after 8 hours of treatment as compared to control. The efficient reduction in the growth of *R. solanacearum* was observed upon treating it with DAPG (Table. 4 and Fig. 2 A-F).

**Table 4:** Live dead assay.

S. No.	Treatments	Percentage of Live cell	No. of Fields
1.	*R. solanacearum*	91	12
2.	*R. solanacearum* + Tetracycline	24	12
3.	*R. solanacearum* + Crude	32	12

### 3.6 Bacterial wilt disease of tomato challenged with *R. Solanacearum*

**Fig. 3:**
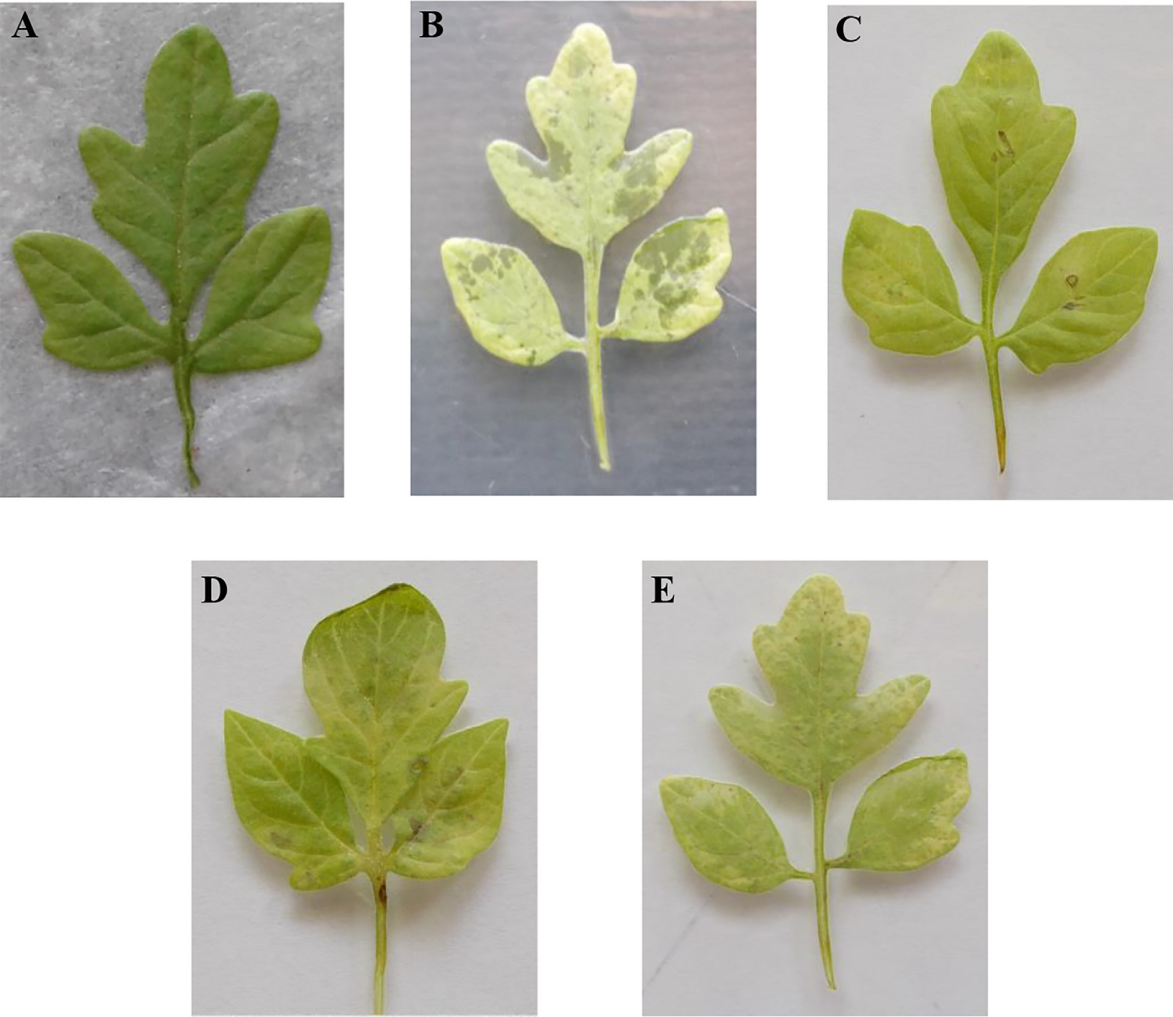
**Detached leaf assay:** A-Control (without inoculation), B-*R. solanacearum* alone only, C- Tetracycline + *R. solanacearum*, D- *P. fluorescens* VSMKU3054 + *R. solanacearum* and E-DAPG + *R. solanacearum.*

The efficacy of *P. fluorescens* VSMKU3054 and DAPG was assessed on three-week-old tomato leaves challenged inoculation with *R. solanacearum* by detached leaf assay. The *P. fluorescens* VSMKU3054 and tetracycline treated with tomato leaves has shown significant reduction of bacterial wilt disease, and the severity was observed on 3rd (59 ± 1 & 58.83 ± 1.04) and 7th day (59.5 ± 0.5 & 59.33 ± 0.76) compared to control. Whereas, the DAPG metabolites from *P. fluorescens* VSMKU3054 was showed wilting symptoms on 3rd day (58.5 ± 1.5) onwards followed by day 7th day (42.12 ± 0.69) tomato leaves were infected, but this experiments were showed incomplete bacterial wilt infection. *R. solanacearum* when treated alone for tomato, the infected leaves got detached on the 3rd day and on 7th day complete wilting occurred in tomato leaves as compared to control (Fig. 3 A-E). The disease scale was measured and the relative AUDPC was calculated and tabulated (Table. 5).

**Table 5:** Detached leaf assay.

Treatments	Disease Scale in AUDPC (%)		
	Day 1	Day 3	Day 7
Control	100 ± 0	100 ± 0	100 ± 0
*R. solanacerum* (RS)	58.33 ± 1.53	33.28 ± 0.75	32.94 ± 0.42
RS + Tetracycline	99 ± 1	58.83 ± 1.04	59.33 ± 0.76
RS + *P. fluorescens*	98.17 ± 1.61	59 ± 1	59.5 ± 0.5
RS + Crude	97 ± 2.65	58.5 ± 1.5	42.12 ± 0.69

### 3.7 Molecular docking analysis

**Fig. 4:**
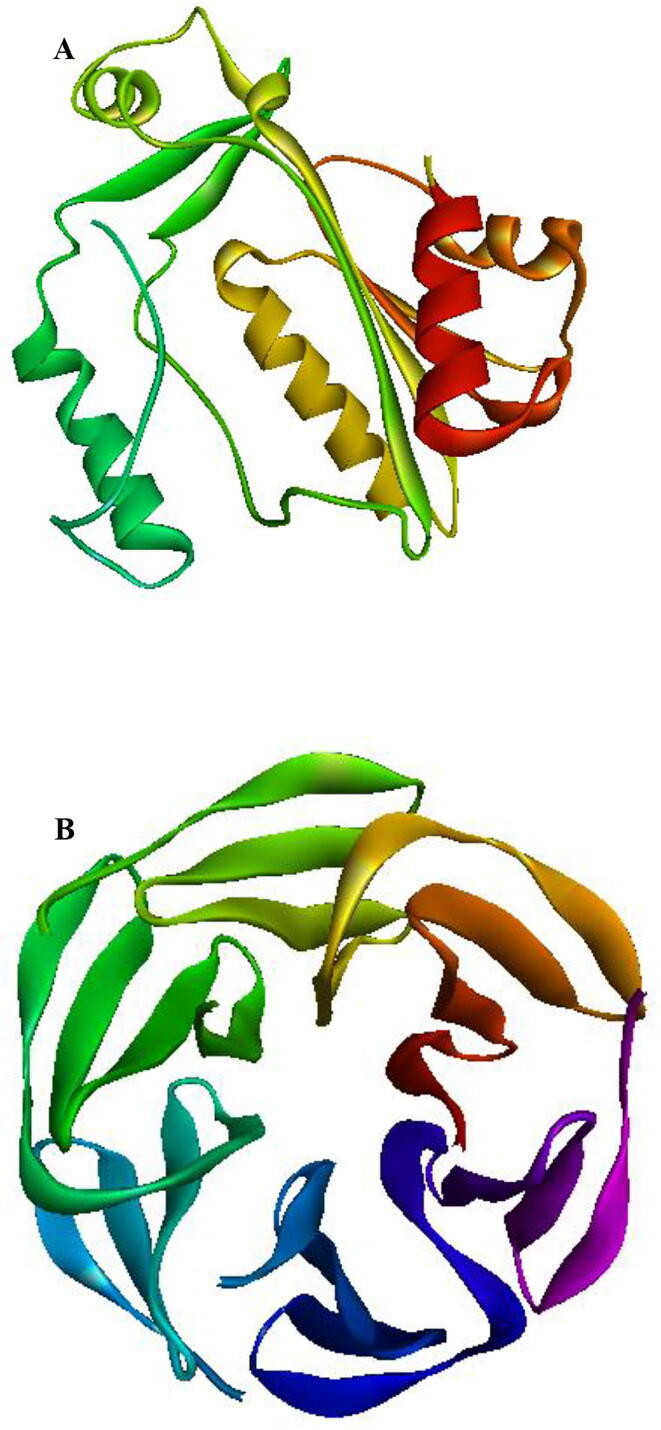

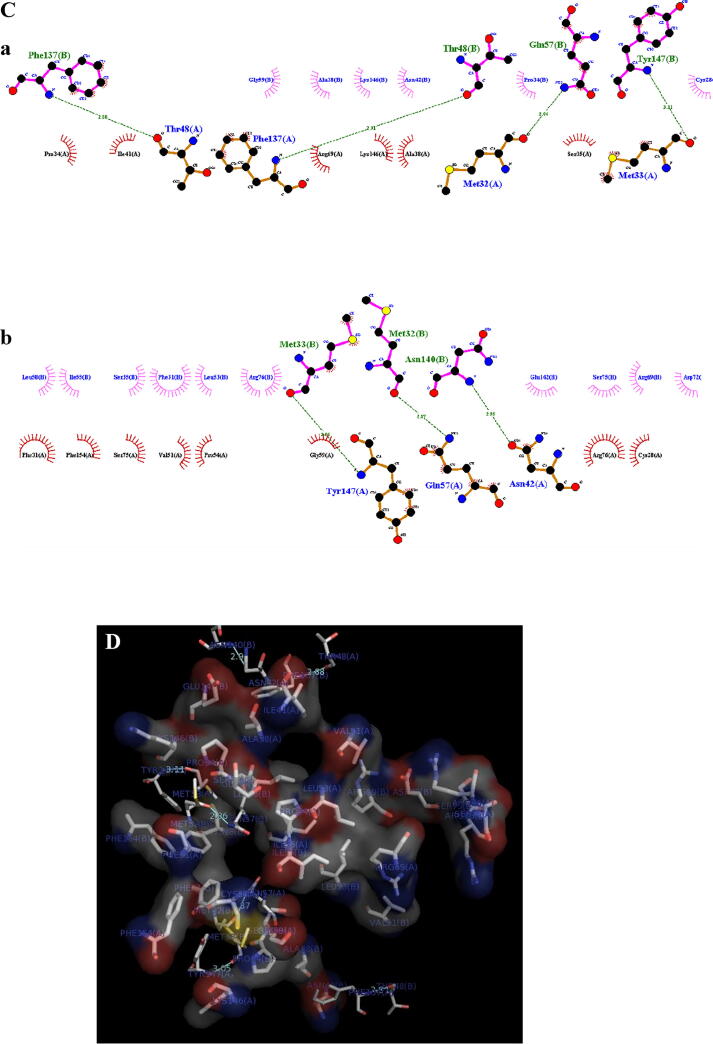
**Molecular Modeling:** A. 3D structure of DAPG gene encoding protein, B. 3D Structure of *R. solanacearum* lectin molecular structure, C-(a&b). 2D View of protein–protein interaction, D. 2D View of protein–protein interaction and E. 3D View of protein–protein interaction.

The 3D structure of DAPG (Fig. 4A) protein was constructed using modular 9v2. The protein was analyzed for its interaction with the Lectin from *R. solanacaum* (Fig. 4B) to understand the mechanism at the molecular level. The details for binding energy, mode of interaction, bond length, and interacting residues were shown in Table 6. Docking results confirmed that the DAPG encoding protease was tightly interacted with Lectin (PDB: 2BT9) with a obligatory free energy of −4.615 kcal/Mol and strong hydrogen bonding through Met 32 and Gly 57 (Fig. 4 C [a, b] & D) of the target protein with a distance of 2.87 Å which causes increased production of proteases.

**Table 6:** Relative binding free energy, mode of interaction and interacting residues.

Ligand	Binding free energy (Kcal/Mol)	Mode of interaction	Residues involved in interaction	Bond length (Å)
Lectin	−4.547	Hydrogen Bond	Met 32 - Gly 57	2.87

## 4 Discussion

Antimicrobial compounds produced by FPs were involved for the control of various bacterial and fungal pathogens. *Pseudomonas* spp (Ayyadurai et al., 2006), *P. aeruginosa* MML2212 (Shanmugaiah et al., 2010), *P. corrugate* GFBP 5454 and *P. mediterranea* GFBP 5447 (Strano et al., 2017), *Pseudomonas* sp VSMKU4036 (Varatharaju et al., 2020, Ansari et al., 2021), and *P. aeruginosa* VSMKU1 (Nithya et al., 2020) are significantly control the growth of different bacterial and fungal plant pathogens by the secretion of diffusible compounds like 2,4-diacetylphloroglucinol (2,4-DAPG), phenazine-1-carboxamide (PCN). Pholoroglucinol assist and act as a signal molecule to induce the expression of pyrrolnitrin biosynthesis. *P. fluorescens* VSMKU3054 produced array of antimicrobial metabolites, and 2,4 diacetylphloroglucinol (2,4 DAPG) is a major encoding gene for the control of various plant pathogens. Moreover DAPG was effectively controlling *R. solanacearum* by *in vitro*.

The presence of DAPG was characterized by TLC, UV & FT-IR spectral studies in DAPG metabolites of our selected isolate VSMKU3054 in comparison with previous reports (Ayyadurai et al., 2006). Brucker et al., (2008) has demonstrated that analysis of DAPG in UV–Visible spectrometer showed a peak around at λ_max_ 270 nm was coincide with that of authentic phloroglucinol. In the present study, GC–MS analysis was indicated that the identical nature of DAPG compounds from *P. fluorescens* VSMKU3054 coincide with that of authentic phloroglucinol (Fig. 1. B,C,D,E and F) (Marchand et al., 2000). Additionally, *M. phaseolina, R. solani, F. oxysporum,* and *F. solani* growth were also significantly retarded the growth of mycelia by metabolites produced from *Pseudomonas* sp in mung bean and sunflower which further enhanced the plant growth (Moin et al., 2020). In the present study, the cell-free culture filtrate and DAPG metabolites of selected potential isolate *P. fluoresens*VSMKU3054 have been observed to reduce the growth of bacterial and fungal pathogens and improved plant growth and thereby protect from plant diseases.

*P. fluorescens* VSMKU3054 produced DAPG metabolites significantly controlled the growth of *R. solanacearum* as compared to tetracycline and untreated cells (Table. 4). This observation in accordance with the previous study showed that the eucalyptus oil (0.07%) treated to *R. solanacearum* had a lower number of live cells when compared to Lemongrass and Palmarosa oil (Paret et al., 2010). Overall we could find out accordance with the previous report and our results showed the potential of partially purified DAPG against plant fungal and bacterial pathogens by ineffectiveness of hyphal tips, modification of plasma membrane, vacuolization and cell content degeneration. Because DAPG have the potential to control wide range of pathogens like fungi and protists by change of membrane integrity and electron transport (Zhou et al., 2012). Further, DAPG and phenazine like compound derived from *Pseudomonas* spp, *P. aeruginosa* MML2212 and *Streptomyces aurantigriseus* VSMGT1014 significantly control *R. solanacearum, Xanthomonas oryzae* and *R. solani* by denucleation, degradation of cytoplasmic content, impairment of mitochondrial function (Zhou et al., 2012, Harikrishnan et al., 2014, Qessaoui et al., 2019). Afroz et al., (2009) demonstrated the detached leaf assay on four different varieties of tomato plants such as Riogarande, Roma, Pusa Ruby, and Pant Bahr upon incubation of 1, 2 and 7 days. Among these four, Roma and Riogarande showed resistance towards bacterial wilt disease in 2 days and symptoms developed on the 7th day. Pusa Ruby and Pant Bahr infected within 1 or 2-days wilt symptoms were observed. In accordance with previous report, our results were coincided for the control of wilt disease of tomato by detached leaf assay by *P. fluorescens* VSMKU30504 and DAPG at 30 µg/ml compared to tetracycline and control (Table. 5, Fig. 3). Polyphenol group of antibiotics have similar structure like DAPG plays an important role for control different plant bacterial and fungal pathogens by suppressing virulent factors of pathogens (Siddiqui and Meon, 2009). In addition to that, our support is molecular docking of DAPG encoding proteins with the listing of *R. solanacearum* showing a strong interaction which indicates the role of DAPG in the control of plant pathogens (Fig. 4). He et al., (2010) has shown that the crystal structure of DAPG is important for biological control of many plant pathogens. Hence, our study strongly demonstrated that *P. fluorescens* VSMKU3054 and DAPG remarkably control *R. solanacearum* by live and dead assay. Further investigations are very much needed for purification and characterization of DAPG from *P. fluorescens* VSMKU3054 form management of wilt disease of Tomato and other fungal diseases (Zhou et al., 2014; Kumar et al., 2005).

## 5 Conclusion

The results of the present study demonstrated that the potential antagonistic *P. fluorescens* produced antimicrobial metabolites of 2,4-DAPG and it was confirmed by different spectral studies. The DAPG reduced population of *R. solanacearum* in cell viability assay and also DAPG have synergistically activity against the bacterial wilt pathogen in the detached leaf assay. Furthermore, molecular docking studies proved the interaction with *R. solanacearum*. Thus, it is concluded that *P. fluorescens* VSMKU3054 can be used as a superior biocontrol agent for bacterial wilt disease management in agricultural fields.

## Declaration of Competing Interest

The authors declared that there is no conflict of interest.
